# Cognitive Adaptation Training for healthcare professionals & relatives: The development of a web-application through User Centered Design

**DOI:** 10.1192/j.eurpsy.2025.1446

**Published:** 2025-08-26

**Authors:** T. van Brouwershaven, B. J. Hoekman, M. de Koning, S. Gangadin, N. Boonstra, L. van der Meer

**Affiliations:** 1 Department of Rehabilitation, Lentis Psychiatric Institute, Zuidlaren; 2 Lectorship digital transformation, Hanzehogeschool Groningen, Groningen; 3 Department of Research, Arkin Institute for Mental Health; 4 Department of Research, Amsterdam UMC, Amsterdam; 5 Department of Psychiatry, UMC Groningen, Groningen; 6 Department of Neuroscience, UMC Utrecht, Utrecht; 7 Department of Research and Innovation, KieN VIP Mental Health Care Services, Leeuwarden; 8 Department of Clinical and Developmental Neuropsychology, University of Groningen, Groningen, Netherlands

## Abstract

**Introduction:**

Cognitive impairments often hinder daily functioning in people with severe mental illness (SMI). Cognitive Adaptation Training (CAT) is an effective psychosocial intervention that reduces this impact. However, barriers such as the two-day training and lack of neuropsychological expertise in some professionals hinder CAT implementation.

**Objectives:**

To make CAT more accessible to healthcare professionals and relatives of people with SMI by developing a web application (House-CAT) that guides users through the intervention.

**Methods:**

For the development, User Centered Design (UCD) is used: a design process where close cooperation with future users (healthcare professionals, relatives, service users) is important to make sure that their needs are met. Although UCD is circular and iterative, three phases can be distinguished: analysis, design, and evaluation. In the analysis phase, users’ needs are identified and translated into design criteria. A testable prototype of House-CAT is created (design phase), followed by implementation and evaluation on efficiency, acceptability, and user-friendliness (evaluation phase).

**Results:**

Three focus groups were conducted in the analysis phase: CAT-experienced professionals (n=5); professionals unfamiliar with CAT (n=4); and relatives (n=5). Further, individual meetings with family members (n=8), and professionals (n=6) were conducted. Design criteria included the app’s ability to (1) support with setting up individual goals; (2) find personalized strategies or tools; (3) improve communication between professionals; (4) encourage cooperation between professionals, relatives, and service users; (5) be available in hybrid form; (6) use simple language. The design- and evaluation phase are currently in process: the web app and results from the evaluation phase will be presented at the conference.

**Conclusions:**

House-CAT should support users in creating individual goals and personalized strategies, stimulate contact between professionals, relatives and service users, and be simple to use.

**Disclosure of Interest:**

None Declared

